# A Spoonful of (Fluorinated) Sugar: The Power of Fluorinated Glycans in the Discovery and Characterization of Glycan–Protein Interactions

**DOI:** 10.1021/acscentsci.4c01206

**Published:** 2024-08-07

**Authors:** Marcello Mercogliano, Flaviana Di Lorenzo

**Affiliations:** † Department of Chemical Sciences, University of Naples Federico II, Naples 80126, Italy; ‡ CEINGE Biotecnologie Avanzate ‘Franco Salvatore’, Naples 80131, Italy

**Figure 1:**
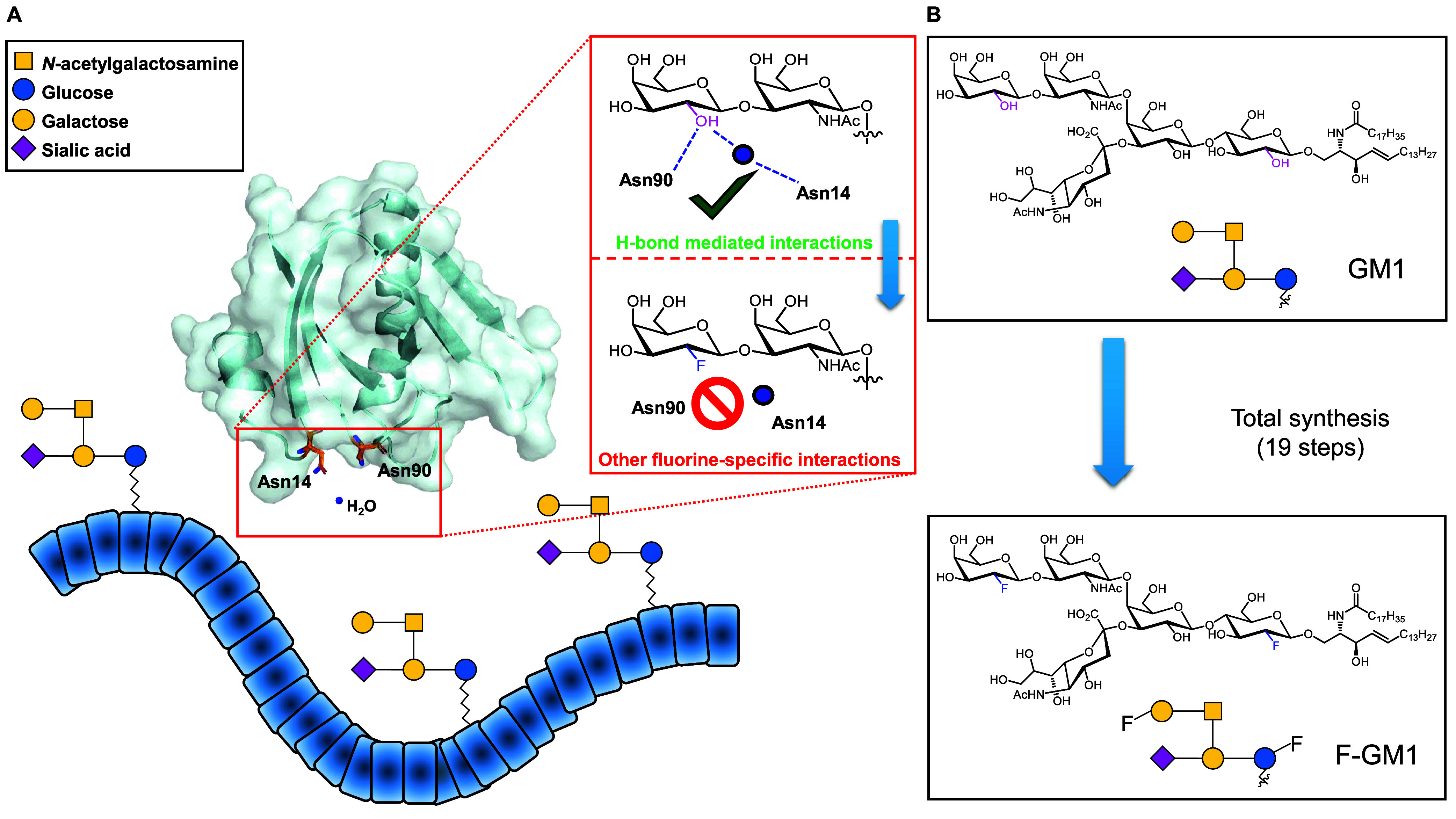
(A) Cartoon of the intestinal epithelial cell line, where native GM1 is exposed and binds with high affinity to the cholera toxin subunit B (CTB_5_). The establishment of H-bonds between the hydroxyl group at position C2 of the terminal galactose of the GM1 oligosaccharide and Asn14 and Asn90 of CTB_5_, which are crucial for a successful interaction, has been reported. Crucially, these H-bonds are not formed when the fluorinated-GM1 derivative (F-GM1) is used for binding to the protein. (B) The structures of both native GM1 and F-GM1 are reported, along with their representations drawn using the Symbol Nomenclature for Glycans (SNFG).

Glycans and glycoconjugates, predominant and vital biomolecules in living systems, are key actors in many biological processes and can either be detrimental or beneficial to the human host. These extremely complex and fascinating molecules are recognized and engaged by several types of bacteria, viruses, and parasites, and targeted by a plethora of toxins. In most cases, toxins are proteins released by bacteria to influence host–pathogen interactions, driving the outcome of these encounters toward the benefit of the pathogen while causing specific lesions and symptoms in the host.^1,2^ An example is the diarrheal disease of cholera, responsible for over 100,000 deaths every year, which is caused by the bacterium *Vibrio cholerae*. This potentially lethal bacterium produces the highly efficient cholera toxin (CT) that, through its binding to the glycolipid GM1 exposed on the plasma membrane of enterocytes, among other cells, exerts its action in the small intestine, causing the extreme intestinal fluid secretion characteristic of cholera patients.^3^ GM1 is a ganglioside composed of a lipid chain (ceramide) and an oligosaccharide (Figure 1), with the latter being the target of the pentameric B subunit of CT (CTB_5_). Previous studies have shown that two key sugars of the GM1 oligosaccharide, galactose (Gal) and sialic acid (Sia), are involved in the interaction. In particular, galactose forms important hydrogen bonds between its C2 hydroxyl group and two asparagine residues (Asn90 and Asn14) of the CTB_5_ protein, resulting in the strongest glycan-protein interaction known. Considering the effects of this interaction on the progression of cholera, it is not surprising that several approaches have been tested to evaluate and confirm the relevance of hydroxyl groups in sugar units for binding to CTB_5_. Nevertheless, direct editing approaches to analyze this hypothesis are still missing in the literature, thus limiting the use of galactose (or other sugar derivatives) as a source for designing selective ligands that could inhibit such dangerous interactions.

From a molecular recognition perspective, ^19^F NMR spectroscopy has tremendous potential to dissect protein–glycan interactions in a fast and reliable manner. Fluorine is an isosteric mimic of the hydroxyl group, but it lacks the capability to act as a hydrogen-bond donor and has low hydrogen-bond acceptor competence. By substituting hydroxyl group(s) with fluorine, it is possible to track how these hydroxyl groups interact with the related recognition protein. This provides information on the binding mode and typically simplifies NMR analysis, making this approach a valuable tool to investigate molecular recognition events from a biomedically promising perspective.^4^

Gilmour and co-workers have already successfully developed fluorinated glycostructures as a molecular editing strategy in pharmaceutical design. In this issue of *ACS Central Science*, they report the total synthesis of a selectively fluorinated GM1 analog (F-GM1), whose binding affinity to CTB_5_ was fully described by NMR and protein crystallography.^1^ Using this unique synthetic procedure, which involved 19 steps, a properly protected Gal(1-3)GalNAc disaccharide was joined to a Sia(2-3)Gal(1-4)Glc trisaccharide. The donor disaccharide was obtained in three steps from a galactose derivative already fluorinated at position 2 via fluorine-directed glycosylation. The trisaccharide was obtained through a sequence of glycosylation and protection/deprotection reactions with high yield and excellent stereocontrol.^1^ The combination of these two sugar motifs produced an advanced intermediate that, after three deprotection steps, resulted in F-GM1. In these final deprotection steps, they observed that the major:minor ratio of two doubly fluorinated conformers present in the final mixture increased from 7:3 to 8:2 to 95:5. The proposed explanation is that the removal of protecting groups causes steric decompression, thus lowering the barrier for conformer interconversion. This was also elegantly confirmed by temperature-varying ^19^F-NMR experiments, which showed that above 40 °C, the NMR signal broadened due to the fast exchange between conformers. This supported that the two species were not diastereoisomers, thereby demonstrating that the final glycosylation reaction achieved exceptional levels of diastereocontrol.^1^

Once the final product was obtained, the binding affinity of F-GM1 for CTB_5_ was again analyzed by ^19^F-NMR and compared to that of the natural compound and other cholera toxin ligands, i.e., the natural GM1, the GM1 pentasaccharide (GM1-PS), and the receptor antagonist *m*-nitrophenyl-galactoside (MNPG), which acted as inhibitors of the F-GM1-CTB_5_ binding. Experimentally, the release of F-GM1 from CTB_5_ in the presence of increasing concentrations of the three inhibitors was plotted as a function of the signal of free F-GM1. The relative affinities of the compounds were then determined based on their IC_50_ values. Except for MNPG, the other inhibitors showed much higher affinity than F-GM1, further demonstrating the importance of having a hydroxyl group at the C2 position of the terminal galactose to establish the H-bonds crucial for interaction with the protein. In addition, these experiments demonstrated that the binding mode and almost all other interactions of F-GM1 with CTB_5_ were preserved.^1^

To gain atomic resolution insights into this “diverse” binding, a high-resolution crystal structure (2.10 Å) of the complex was prepared. Interestingly, Gilmour and co-workers showed that F-GM1 binds to CTB_5_ in the same manner as the native GM1, but with one crucial difference in the terminal fluorinated galactose; i.e., the substitution of the hydroxyl group with fluorine led to a change in the sugar ring conformation. As a result, the quasi-axial arrangement of the C2-fluorine bond enabled the establishment of other interactions involving the carbonyl group of the adjacent GalNAc unit and the Ans90 residue of the CTB_**5**_ protein. This arrangement disrupted crucial H-bonds and confirmed that only the intended H-bonds between the fluorinated ligand and the protein were eliminated.^1^

Overall, this is an extremely interesting study that provides compelling evidence of the utility of hydroxyl-by-fluorine substitution in glycobiology. Smart use of mono- and polyfluorinated glycan structures has strongly emerged on the scientific panorama due to their capability to behave as excellent bioisosteres of 2-deoxy sugars and their promising properties for enhancing pharmacokinetic profiles and metabolic stability.^5^ Nevertheless, methods for the preparation of fluorinated oligosaccharides are still underdeveloped. Therefore, the work by Gilmour and co-workers represents a two-fold success. They developed a highly effective synthetic protocol to meet the clear demand for pharmaceutical candidates as well as molecular probes. Additionally, they delivered fluorinated gangliosides that not only will simplify structural delineation of such a crucial protein–ligand interaction but also provide new molecules with high potential and tremendous implications for probing glycan signal transduction, drug discovery, and vaccine development.
